# Once an Impression Manager, Always an Impression Manager? Antecedents of Honest and Deceptive Impression Management Use and Variability across Multiple Job Interviews

**DOI:** 10.3389/fpsyg.2017.00029

**Published:** 2017-01-24

**Authors:** Nicolas Roulin, Joshua S. Bourdage

**Affiliations:** ^1^Asper School of Business, University of ManitobaWinnipeg, MB, Canada; ^2^Department of Psychology, University of CalgaryCalgary, AB, Canada

**Keywords:** impression management, faking, personality, individual differences, variability, competitive worldviews, interview, personnel selection

## Abstract

Research has examined the antecedents of applicants' use of impression management (IM) tactics in employment interviews. All existing empirical studies have measured IM in one particular interview. Yet, applicants generally interview multiple times for different positions, and thus have multiple opportunities to engage in IM, before they can secure a job. Similarly, recent theoretical advances in personnel selection and IM research have suggested that applicant behaviors should be considered as dynamic and adaptive in nature. In line with this perspective, the present study is the first to examine the role of individual differences in both applicants' use of IM tactics and the variability in IM use across multiple interviews. It also highlights which honest and deceptive IM tactics remain stable vs. vary in consecutive interviews with different interviewers and organizations. Results suggest that applicants high in Extraversion or core self-evaluations tend to engage in more honest self-promotion but do not adapt their IM approach across interviews. In contrast, applicants who possess more undesirable personality traits (i.e., low on Honesty-Humility and Conscientiousness, but high on Machiavellianism, Narcissism, Psychopathy, or Competitive Worldviews) tend to use more deceptive IM (and especially image creation tactics) and are also more likely to adapt their IM strategy across interviews. Because deceptive IM users can obtain better evaluations from interviewers and the personality profile of those users is often associated with undesirable workplace outcomes, this study provides additional evidence for the claim that deceptive IM (or faking) is a potential threat for organizations.

## Introduction

Impression management (IM), or applicants' attempts to influence interviewers' evaluations and decisions through a variety of tactics, has been extensively studied in personnel selection research. We know, for instance, that a large majority of applicants engage in IM (Stevens and Kristof, 1995; Ellis et al., 2002), that IM tactics can be honest but also deceptive (Levashina and Campion, 2007), that interviewers are generally incapable of accurately detecting such tactics (Roulin et al., 2015), and that IM can largely impact interviewers' evaluations (Barrick et al., 2009). A number of theoretical models (e.g., Bozeman and Kacmar, 1997; Levashina and Campion, 2006) and empirical studies (e.g., Kristof-Brown et al., 2002; Fell et al., 2016) have also highlighted potential predictors of IM use, including stable personality traits, abilities, or cultural factors.

So far, empirical studies have only examined IM use at a single point in time (i.e., in one particular interview). This line of research is a necessary starting point to better understand the antecedents, processes, and outcomes associated with applicant IM. Yet, in most cases, applicants on the job market likely apply for multiple positions and interview multiple times before they receive (and eventually accept) a job offer. As such, they usually have multiple opportunities to engage in IM behaviors. However, extant IM research has not studied how stable vs. variable IM use is. On the one hand, it is possible that applicants engage in one particular IM strategy during their first interview and then systematically use it in subsequent interviews. Alternatively, applicants may adapt their IM strategy from one interview to the next. They may decide to engage in more (or less) IM, or to adapt some of their IM tactics while keeping other more constant. This would be in line with recent theoretical developments in personnel selection (Bangerter et al., 2012) and IM in particular (Roulin et al., 2016), which describe applicants' behaviors as dynamic and adaptive in nature, and call for more research on applicants' behaviors across multiple selection encounters.

Many theoretical models of IM (or faking) in the interview suggest that applicants need to have the capacity, the willingness/motivation, and the opportunity to engage in IM (Levashina and Campion, 2006; McFarland and Ryan, 2006; Roulin et al., 2016). We argue that applicants' capacity, willingness, and opportunity to engage in IM may vary from one interview to the next, thus influencing their actual use of IM tactics. First, applicants' capacity to use IM tactics may increase as they learn from (successful or unsuccessful) interviewing experiences (Roulin et al., 2016). For instance, an applicant may use some specific IM tactics in initial interviews, accumulate knowledge about the effectiveness of these tactics (i.e., which ones led to positive reactions from interviewers), and subsequently adapt to use only the most effective tactics. Second applicants' willingness to use IM may evolve depending on the success or failure of past IM attempts or the characteristics of the interviewer. For instance, an applicant may shy away from IM in initial interviews and be unsuccessful, prompting them to be more motivated or willing to use IM in the future. Or they may end up with an interviewer who heavily uses IM themselves, thereby signaling it as appropriate. Finally, applicants may have more or less opportunities to use IM depending on the format of the interview or the type of questions asked by the interviewer (Van Iddekinge et al., 2007; Levashina et al., 2014). As such, the same applicant interviewing multiple times for different positions, in different organizations, and with different interviewers will have different opportunities to use IM.

It is likely that applicants' use of IM changes from one interview to the next, but not all applicants may adapt the same way. Indeed, Roulin et al. (2016) suggest that there may be individual differences associated with IM adaptation and thus variability in IM use. Yet, although there is some research examining the applicant characteristics associated with IM use, no study has explored factors associated with variability in IM use.

In summary, the present research is an initial attempt to investigate IM use across multiple interviews. It contributes to the interview IM literature in three major ways. First, it provides additional evidence about which individual characteristics are associated with honest and deceptive IM use in general, using a more robust approach to measure IM (i.e., the average IM use across multiple interviews instead of in only one encounter). Second, this study represents the first examination of applicants' use of IM tactics in several consecutive interviews with different interviewers and organizations. It thus highlights how stable vs. variable honest and deceptive IM tactics are. Finally, this study explores a range of stable individual characteristics of applicants (e.g., personality, Competitive Worldviews, self-monitoring) to identify which ones are associated with IM variability (and for which type of IM tactics). In other words, it highlights what kinds of applicants are more likely to adapt their IM strategy across interviews.

## Honest and deceptive IM in job interviews

### Understanding IM in interviews

IM use can have a large impact on interviewers' ratings of interview performance (Barrick et al., 2009). Therefore, understanding who is engaging in IM can tell us about the extent to which IM is problematic. For instance, it can inform as to whether IM is (a) a universally adopted behavior, (b) used by those who are very achievement oriented, and who later can be strong job performers, or (c) whether it is the tool of unqualified or manipulative individuals, who would later be a detriment to the organization.

Researchers have emphasized that applicants must be comfortable using IM before engaging in such behaviors (Kacmar et al., 1992) and may also vary in their willingness or capacity to engage in such tactics (Levashina and Campion, 2006). This suggests that IM is unlikely to be engaged in universally (or to the same extent) by all applicants. Moreover, studies have started to explore the other alternatives by examining individual differences associated with IM use. For instance, researchers have shown that IM use can be associated with the personality traits of Extraversion (Kristof-Brown et al., 2002), Conscientiousness and Agreeableness (Peeters and Lievens, 2006), or with self-monitoring (Higgins and Judge, 2004).

### Honest vs. deceptive IM

Although those studies have been informative, most of them failed to differentiate honest from deceptive attempts of applicants (Levashina et al., 2014). The distinction between honest and deceptive IM is an important theoretical and practical distinction. Although applicants can manage their impressions in a variety of ways, organizations want to know if applicants try to create an impression of competence and fit by emphasizing positive true past experiences and values similarities with the organization (honest IM) or pretend to have qualifications, experiences, or fit that they do not possess (deceptive IM). Moreover, deceptive IM (perhaps unlike honest IM) may introduce a source of inaccuracy in interviewers' evaluations (Posthuma et al., 2002; Levashina and Campion, 2006). However, although honest and deceptive IM seem fundamentally different in nature and are likely to have different antecedents, most of the extant research has relied on self-reported measures of IM that confound the two, or leave the nature ambiguous (Higgins and Judge, 2004; Levashina et al., 2014). Other studies have relied on coder ratings of IM, which may further obfuscate relationships, as studies show that coders generally do not accurately identify honest from deceptive IM use (Roulin et al., 2015). As a result, it is unclear whether the findings described above depicted relationships with honest IM, deceptive IM, or both.

In the present study, we therefore focus on antecedents of both honest and deceptive IM. Guided by the most recent work in the area, we investigate three honest IM tactics and four deceptive IM tactics. The three honest IM behaviors are measured in the Honest Interview Impression Management scale by Roulin et al. (2014), which includes honest self-promotion, honest ingratiation, and honest defensive IM. On the other hand, we measure the four deceptive tactics captured in the Interview Faking Behavior scale developed and validated by Levashina and Campion (2007), and encompasses slight image creation, extensive image creation, image protection, and deceptive ingratiation. We present detailed definitions, and sample items, for each type of IM tactic in Table 1.

**Table 1:** **Definitions and example items for honest and deceptive IM tactics**.

**IM tactics**	**Definitions**	**Sample items**
Honest self-promotion	Pointing out one's actual past experiences or accomplishments and describing one' actual job-related abilities or skills in an attractive way	I made sure the interviewer was aware of my skills and abilities
Honest ingratiation	Highlighting values that one shares with the interviewer or organization, or genuinely praising the interviewer or the organization	I found out about values and goals that I shared with the organization, and made sure to emphasize them
Honest defensive IM	Using excuses, apologies, or justifications to repair one's image when threatened by negative questions of concerns from the interviewer	I gave reasons why I felt I benefited positively from a negative event I was responsible for
Slight image creation	Embellishing, overstating, tailoring, or enhancing one's qualifications or experiences to appear more qualified for the position or a better fit with the organization	I inflated the fit between my values and goals and the values and goals of the organization
Extensive image creation	Inventing, constructing, or borrowing qualifications or experiences to appear more qualified for the position or a better fit with the organization	I told fictional stories prepared in advance of the interview to best present my credentials
Deceptive ingratiation	Expressing false beliefs, values, or attitudes to appear similar to the interviewer or organization, or insincerely praising or complimenting the interviewer or the organization	I complimented the organization on something, however insignificant it may actually be to me
Image protection	Omitting, hiding, or distancing oneself from negative events in one's past to defend one's image of a good candidate	I covered up some “skeletons in my closet”

*Definition and items adapted from Levashina and Campion (2007) and Roulin et al. (2014)*.

## Individual differences predicting IM use

### Theoretical antecedents of IM use

In their model delineating the antecedents of deceptive IM use, Roulin et al. (2016) propose that applicants are more motivated to engage in such tactics if they perceive the competition for the job to be intense and have positive attitudes toward faking. Moreover, they argue that individual differences are the main drivers of such perceptions and attitudes. According to Roulin et al. (2016), perceived competition largely derives from applicants' Competitive Worldviews, that is, their stable beliefs that the world is a competitive jungle where people fight to obtain scarce resources (Duckitt et al., 2002). Moreover, attitudes toward faking are more positive if applicants possess a “darker” personality profile (e.g., high in Machiavellianism or Narcissism; Jonason and Webster, 2010), but more negative if applicants value honesty and integrity. Levashina and Campion's (2006) model offers a complementary perspective by highlighting the potential role of self-monitoring and the Big-Five personality traits in applicants' use of IM. They propose that high self-monitors, who are better able to adjust their behaviors and control their expressions, should be more willing to engage in deceptive IM during an interview. Moreover, they suggest that extroverted applicants, who are more comfortable in social interactions and thus have more opportunities to influence others and deceive, will be more willing to engage in faking during an interview. The same argument can arguably be used for honest IM, that is, extraverted applicants are also more likely to highlight their true qualifications and thus engage in more honest IM. Alternatively, Levashina and Campion (2006) suggest that conscientious applicants, who are better prepared for their interview, and agreeable applicants, who are more likely to adhere to social norms, should be more honest and less willing to engage in faking.

### Existing empirical evidence

There is already some empirical support for the propositions of both Roulin et al. (2016) and Levashina and Campion (2006). For instance, studies focused on deceptive IM found that applicants low on Honesty, but high on Machiavellianism, Narcissism, self-monitoring, and Competitive Worldviews are more likely to use image creation in their interview (Levashina and Campion, 2007; Hogue et al., 2013; Roulin and Krings, 2016). Furthermore, Bourdage et al. (2015) examined both honest and deceptive IM. They found that deceptive IM was associated with the HEXACO personality traits of low Honesty-Humility, low Conscientiousness, as well as high Competitive Worldviews. In contrast, honest IM was only associated with high Extraversion. However, they did not explore how other factors that have been associated with deceptive IM, such as the dark triad of personality, relate to honest IM use.

### Examining antecedents of IM use across multiple interviews

Building on Levashina and Campion's (2006) and Roulin et al. (2016) models, the first objective of the present study is to replicate and extend the findings presented above. Moreover, we propose to examine the role of an additional stable characteristic of applicants that could be associated with IM: core self-evaluations. Core self-evaluations capture individuals' positive self-concept perceptions (Judge et al., 2003), and have been associated with a number of positive workplace outcomes, such as job satisfaction and work performance (Judge and Bono, 2001). As such, it is likely that applicants with positive core self-evaluations will be confident in their qualifications, and thus engage in more honest (but not deceptive) IM to highlight them. Altogether, we expect the following relationships between individual differences and honest and deceptive IM use:

*Hypothesis 1*: Conscientiousness (H1a), Agreeableness (H1b), Extraversion (H1c), and core self-evaluations (H1d) are positively associated with honest IM use.
*Hypothesis 2*: Conscientiousness (H2a), Agreeableness (H2b), and Honesty-Humility (H2c) are negatively associated with deceptive IM use, while Extraversion (H1d), Machiavellianism (H2e), Narcissism (H2f), Psychopathy (H2g), self-monitoring (H2h), and Competitive Worldviews (H2i) are positively associated with deceptive IM use.

In order to obtain a more robust estimation of the relationship between individual differences and IM use in this study, we measure the average use of each IM tactic across multiple interviews instead of relying on a measure at only one point in time.

## Individual differences predicting variability in IM use

### Applicants' adaptations in IM use

The personnel selection process is, by its very nature, a dynamic and adaptive process. In their framework for personnel selection derived from signaling theory, Bangerter et al. (2012) highlight the evolution of selection systems over time. For instance, they describe how applicants learn about the types of questions asked by interviewers and the best strategies to impress them, thus forcing organizations to adapt their interviewing process to limit applicants' opportunity to fake. Building on that framework, Roulin et al. (2016) proposed a dynamic model of applicant faking. This model, whose principles can be applied to IM in general, explains how applicants adapt their use of IM tactics from one interview to the next. More precisely, like other IM models (e.g., Levashina and Campion, 2006; McFarland and Ryan, 2006), Roulin et al.'s (2016) model describes motivation and capacity as two immediate antecedents of applicants' use of IM. But it also illustrates how applicants' capacity and motivation to engage in IM can evolve.

### Self-monitoring, CWs, locus of control, and IM adaptations

Applicants' capacity to effectively engage in IM changes as they accumulate experience with interviews (Roulin et al., 2016). With each interview, applicants can learn from their successful or unsuccessful attempts and realize which IM tactics are more (or less) effective. Moreover, they can also develop their ability to detect and interpret information provided by the organization to use the right tactic at the right moment. For instance, they may realize that one type of ingratiation tactic (e.g., highlighting similarities with the interviewer) is more effective than another (e.g., praising the organization) for them, or get better at identifying the right moment to use this tactic in an interview. Yet, Roulin et al. (2016) propose that not all applicants can increase their capacity to use IM tactics in the same way. More precisely, they suggest that self-monitoring (i.e., individuals' ability to manage their behaviors and evaluate others' reactions to such behaviors; Snyder, 1974) facilitates applicants' learning process, and thus their adaptations in IM use.

Applicants' willingness or motivation to use IM also changes as they interpret the outcome of their interview and the eventual feedback they received from the organization (Roulin et al., 2016). If their initial attempt to secure a job was unsuccessful, they may perceive their lack of success as a signal that their initial strategy was insufficient to outperform other candidates for the job, and will feel pressured to adapt their use of IM to increase their chances of success in subsequent interviews. For instance, applicants who engaged in honest IM only (or limited deceptive IM) may be more motivated to engage in deceptive IM in their next interview. Alternatively, applicants who extensively engaged in deceptive IM may adapt by engaging in more honest IM. Yet, Roulin et al. (2016) propose that not all applicants perceive the pressure to adapt their use of IM tactics in the same way. They suggest that applicants with higher Competitive Worldviews (i.e., who perceive the world like a competitive jungle; Duckitt et al., 2002) or with an internal locus of control (i.e., who believe that they can control their external environment; Rotter, 1966) are particularly sensitive to interview outcomes. Those individuals are more likely to interpret a (perceived) lack of success as a signal that they should (and can) “up their game” to outperform other applicants, and are thus more inclined to adapt their use of IM. Consistent with the propositions of the Roulin et al.'s (2016) model, we thus anticipate that applicants higher on self-monitoring, Competitive Worldviews, and core-self-evaluations (which incorporate internal locus of control; Judge and Bono, 2001) will be more prone to adapt their use of IM tactics.

### Personality and IM adaptations

Although Roulin et al. (2016) only describe personality traits (e.g., honesty or the dark triad of personality) as the source of stable attitudes toward IM, we argue that applicants' personality traits may also be associated with different adaptive mechanisms. For instance, individuals high on Honesty-Humility prefer interpersonal relations to be genuine and sincere rather than based on manipulation and deceit (Ashton et al., 2014). Even when they have the opportunity to cheat or take advantage of others (Hilbig and Zettler, 2015), or to take risks associated with clear potential gains (Weller and Thulin, 2012), high Honesty-Humility people generally refrain from doing so. Such individuals are thus less likely to rethink their motivation to using IM tactics, and are thus less likely to adapt their IM behaviors from one interview to the next, even if they see the potential benefits of such a strategy.

In sharp contrast, individuals low in Honesty-Humility, and high on the dark triad of personality (i.e., Machiavellianism, Narcissism, and Psychopathy) tend to favor short-term, strategic behaviors in interpersonal relations (Jonason and Schmitt, 2012), adapt their behaviors to extract the most resources from their interaction partners (O'Boyle et al., 2012), and do not hesitate to manipulate or exploit others to achieve their objectives (Jonason et al., 2012; Hilbig and Zettler, 2015). We thus anticipate that applicants high on the dark triad of personality will not hesitate to adapt their willingness or motivation to use IM if they believe that it may help them get the job, and thus adapt their use of IM tactics. Overall, we expect the following relationships between individual differences and variability in IM use:

*Hypothesis 3*: self-monitoring (H3a), Competitive Worldviews (H3b), core self-evaluations (H3c), Machiavellianism (H3d), Narcissism (H3e), and Psychopathy (H3f) are positively associated with variability in IM use, whereas Honesty-Humility (H3g) is negatively associated with variability in IM use.

## Method

### Sample

The sample was composed of 80 senior business students completing a total of 448 interviews. These students were involved in the co-operative education program in a Canadian university. All participants were interviewing with one or more local organization(s) to obtain a 3-months-long job placement. Interviewees were 20.57 years old on average (*SD* = 1.38), mostly female (73%), Caucasian (47%), or Asian (41%), were typically in the third year of their degree (*M*_year_ = 3.10), and had participated in an average of 9.40 (*SD* = 7.44) job interviews prior to the study. All participants interviewed with professional interviewers from local organizations active in a variety of industries (e.g., banking, auditing and accounting, insurance, transportation, agriculture, or energy). We obtained demographic information from a subsample of 82 interviewers involved in the present study. They were 34.34 years old on average (*SD* = 9.02), mostly female (67%), in managerial roles (63%), and had extensive experience conducting job interviews (i.e., they conducted on average 174.56 interviews in their career, *SD* = 299.39).

### Procedure

All students were involved in the following recruitment and interviewing process organized by the business school's co-operative education program: First, students submit their resume and application to one or multiple organizations they are interested in working for. Organizations then review applications and invite their preferred candidates for a 30-min-to-1-h interview. Interviews take place in interview rooms on campus during a 2-week period. The content of the interviews, the level of formality or structure involved, and the type of questions asked are decided by the organization and/or their interviewer(s). At the end of the interview period, organizations make job placement offers to their top candidates.

Participants were contacted approximately 2 weeks prior to their first interview and completed an online questionnaire containing all the individual difference variables (e.g., personality) and demographic information. The day of their first interview, they received a printed (or electronic) package with copies of the IM measure (i.e., a 28-item questionnaire about their use of IM tactics—see *Measures* section below). They were instructed to complete one copy after each interview, and to bring (or email) completed forms to the co-operative education office or to a research assistant. They were also informed that their responses would not be shared with interviewers or the co-operative education team. In total, we obtained a total of 448 completed IM forms (i.e., students participated in one to fifteen interviews, with 5.80 interviews on average). At the end of the interview process, participants received a gift card equivalent to $10 for completing the online part and $5 for each post-interview IM form completed.

### Measures

In line with the tradition in interview IM research (e.g., Stevens and Kristof, 1995; Levashina and Campion, 2007), and because coders cannot effectively differentiate honest from deceptive IM (Roulin et al., 2015), we rely on self-reports in the present study.

#### HEXACO personality

Personality was measured using self-reports on the 60-item HEXACO-PI-R (Lee and Ashton, 2004; Ashton and Lee, 2009). The HEXACO model of personality measures the six personality dimensions of Honesty-Humility, Emotionality, Extraversion, Agreeableness, Conscientiousness, and Openness to Experience. Each of the six personality dimensions is measured using 10-items with a 5-point Likert scale from 1 (*Strongly disagree*) to 5 (*Strongly agree*). A sample Conscientiousness item is “I plan ahead and organize things, to avoid scrambling at the last minute.” The internal consistency reliability of all of the factors was acceptable, with alphas ranging from 0.71 to 0.80.

#### Dark triad of personality

We measured the dark triad of personality using the 12-item “Dirty Dozen” scale by Jonason and Webster (2010). Reliability coefficients were good for all three traits: (*alphas* = 0.75–0.83). Example items included “I tend to manipulate others to get my way” (Machiavellianism), “I tend to want others to admire me” (Narcissism), or “I tend to be unconcerned with the morality of my actions” (Psychopathy). Responses were indicated on a 5-point rating scale, from 1 (*strongly disagree*) to 5 (*strongly agree*).

#### Core self-evaluations

We used the 12-item (*alpha* = 0.86) core self-evaluations scale (Judge et al., 2003). One example item is “I am confident I get the success I deserve in life.” Responses were indicated on a 5-point rating scale, from 1 (*strongly disagree*) to 5 (*strongly agree*).

#### Competitive worldviews

We used the 20-item (*alpha* = 0.90) Competitive Jungle Social World View scale (Duckitt et al., 2002). One example item is “it's a dog-eat-dog world where you have to be ruthless at times.” Responses were indicated on a 5-point rating scale, from 1 (*strongly disagree*) to 5 (*strongly agree*).

#### Self-monitoring

We used the self-monitoring measure developed by Snyder (1974), with 25 true-false statements (*alpha* = 0.69). One example item is “I would probably make a good actor.”

#### Impression management

IM was measured using short versions of the Honest Interview Impression Management (Roulin and Bourdage, 2016) and Interview Faking Behavior (Levashina and Campion, 2007). This 28-item measure includes self-reports of honest self-promotion, honest ingratiation, honest defensive IM, slight image creation, extensive image creation, deceptive ingratiation, and image protection (with 4 items for each IM tactic). Participants were asked to what extent they used each of the tactics. Responses were made on a 5-point Likert scale from 1 (*To no extent*) to 5 (*To a very great extent*). Internal consistency reliabilities ranged from 0.67 (honest ingratiation) to 0.89 (honest self-promotion).

## Results

### IM use and variability

We computed three IM indicators at the interviewee level using our data. First, we computed the average IM use across all interviews (independently for the 7 types of IM tactics). We present the descriptive statistics as well as the correlations between our individual differences and the IM use indicators in Table 2. The most prevalent IM tactics were honest self-promotion and honest ingratiation, whereas extensive image creation and image protection were less commonly used.

**Table 2:** **Means, standard deviations, and correlations for IM use and stable individual characteristics**.

		***M***	***SD***	**1**	**2**	**3**	**4**	**5**	**6**	**7**	**8**	**9**	**10**	**11**	**12**	**13**	**14**	**15**	**16**	**17**	**18**	**19**	**20**	**21**	**22**
**DEMOGRAPHIC VARIABLES**																									
1.	Year in program	3.10	0.96	–																					
2.	Past interview experience	9.40	7.44	0.39	–																				
3.	Number of interviews	5.80	3.16	−0.06	0.11	–																			
**INDIVIDUAL DIFFERENCES**																									
4.	Agreeableness	3.16	0.62	0.02	0.27	0.08	(0.80)																		
5.	Conscientiousness	3.80	0.54	0.08	−0.09	0.10	0.05	(0.77)																	
6.	Emotionality	3.38	0.64	−0.10	−0.12	0.21	−0.09	0.08	(0.80)																
7.	Extraversion	3.52	0.55	−0.03	−0.16	−0.17	0.18	0.22	−0.21	(0.79)															
8.	Honesty-humility	3.31	0.65	0.05	0.03	−0.07	0.44	0.22	0.04	−0.13	(0.79)														
9.	Openness	3.24	0.58	0.10	0.17	−0.29	0.24	0.10	−0.27	0.17	0.30	(0.71)													
10.	Psychopathy	2.22	0.74	−0.09	−0.16	−0.05	−0.40	−0.27	−0.27	−0.15	−0.41	−0.14	(0.75)												
11.	Machiavellianism	2.40	0.91	−0.07	−0.20	−0.01	−0.35	−0.10	−0.09	0.11	−0.63	−0.32	0.60	(0.83)											
12.	Narcissism	3.07	0.75	−0.01	−0.19	0.15	−0.42	−0.09	0.14	0.06	−0.58	−0.16	0.41	0.62	(0.75)										
13.	Core self-evaluations	3.53	0.56	0.11	−0.06	0.03	0.17	0.45	−0.39	0.66	0.04	0.11	−0.27	−0.11	−0.21	(0.86)									
14.	Competitive Worldviews	2.15	0.54	−0.05	−0.09	−0.06	−0.41	−0.27	−0.17	−0.03	−0.64	−0.20	0.68	0.60	0.47	−0.21	(0.90)								
15.	Self-monitoring	0.53	0.19	−0.07	−0.16	−0.04	−0.15	0.00	−0.07	0.28	−0.38	−0.04	0.12	0.33	0.27	0.05	0.22	(0.69)							
**IMPRESSION MANAGEMENT USE**																									
16.	Honest self-promotion	4.02	0.64	−0.04	0.03	−0.06	−0.05	0.11	−0.08	0.34	−0.16	0.04	0.03	−0.03	0.04	0.20	0.14	0.13	(0.89)						
17.	Honest ingratiation	3.53	0.79	−0.10	−0.02	−0.02	0.09	−0.11	−0.07	0.20	−0.13	0.14	−0.02	−0.02	0.04	0.13	0.04	0.06	0.26	(0.67)					
18.	Honest defensive IM	2.77	0.91	0.04	−0.13	−0.17	0.13	0.19	−0.05	0.15	−0.05	0.16	0.01	0.08	0.08	0.07	0.16	0.01	0.39	0.25	(0.87)				
19.	Slight image creation	2.04	0.96	−0.18	−0.17	−0.20	−0.13	−0.31	−0.11	0.15	−0.39	0.05	0.37	0.43	0.26	−0.09	0.45	0.18	0.02	0.14	0.21	(0.88)			
20.	Extensive image creation	1.38	0.56	−0.21	−0.07	−0.19	−0.03	−0.34	−0.01	0.04	−0.25	0.09	0.30	0.31	0.23	−0.17	0.39	0.10	−0.13	0.00	0.15	0.62	(0.83)		
21.	Deceptive ingratiation	2.99	0.94	−0.26	−0.19	0.07	−0.12	−0.18	−0.01	0.21	−0.36	−0.07	0.09	0.17	0.17	0.10	0.22	0.17	0.25	0.36	0.08	0.38	0.09	(0.85)	
22.	Image protection	1.54	0.66	−0.26	−0.05	−0.08	−0.10	−0.20	0.05	0.20	−0.30	0.04	0.15	0.26	0.23	−0.08	0.30	0.13	0.16	0.15	0.18	0.52	0.62	0.44	(0.70)

*N = 80. Reliability coefficients (i.e., Cronbach's alphas) are presented in parentheses. Values = |0.30| are significant at p < 0.01 (two-tailed), values = |0.26| are significant at p < 0.01 (one-tailed), values = |0.25| are significant at p < 0.05 (two-tailed), and values = |0.19| are significant at p < 0.05 (one-tailed)*.

Then, we obtained two indicators of variability in IM use, by computing (a) the standard deviation across all interviews and (b) the coefficient of variation (i.e., *SD/Mean*) across all interviews. The standard deviation is a traditional indicator of variability, and represents an absolute measure of change in IM use. Yet, it is proportional to (and can thus be impacted by) the average IM use. In contrast, the coefficient of variation represents a measure of relative variability, and thus allows for direct comparisons between IM tactics that are not impacted by the average IM use. We thus compared the variability of the seven IM tactics using the coefficient of variation indicators. We observed the most variability for honest defensive IM, with a 95% Confidence Interval of [0.20, 0.27], slight image creation [0.18, 0.24], honest ingratiation [0.16, 0.21], deceptive ingratiation [0.16, 0.21], and image protection [0.14, 0.21], but far less variability for honest self-promotion [0.10, 0.14] and extensive image creation [0.11, 0.17]. We present the descriptive statistics for IM variability, and the correlations between the individual differences and the two indicators of variability in Table 3.

**Table 3:** **Variability in IM use across multiple interviews and relationships with stable individual characteristics**.

	**Variability measured as standard deviation**							**Variability measured as coefficient of variation**						
	**HON SP**	**HON ING**	**HON DEF**	**SIC**	**EIC**	**DEC ING**	**IP**	**HON SP**	**HON ING**	**HON DEF**	**SIC**	**EIC**	**DEC ING**	**IP**
*Mean*	0.46	0.66	0.56	0.41	0.21	0.52	0.28	0.12	0.18	0.23	0.21	0.14	0.19	0.17
*SD*	0.28	0.67	0.33	0.29	0.20	0.27	0.27	0.09	0.12	0.15	0.14	0.12	0.11	0.15
**CORRELATIONS WITH**…														
Year in program	−0.02	0.07	−0.02	−0.23	−0.09	0.06	−0.12	−0.03	0.07	−0.08	−0.13	−0.04	0.20	−0.04
Interview experience	0.17	0.15	0.01	0.06	0.08	0.10	−0.01	0.10	0.17	0.07	0.14	0.05	0.21	−0.07
Number of interviews	0.31	0.03	0.27	0.19	0.02	0.20	−0.02	0.31	0.19	0.37	0.31	0.01	0.20	0.00
Agreeableness	−0.18	0.01	−0.09	−0.05	0.07	0.11	−0.19	−0.10	0.02	−0.09	−0.04	0.04	0.15	−0.23
Conscientiousness	−0.10	0.04	0.14	−0.15	−0.27	0.03	−0.05	−0.13	0.14	−0.06	0.02	−0.20	0.11	0.03
Emotionality	−0.05	0.01	0.09	0.03	−0.05	0.08	0.04	0.04	0.06	0.13	0.10	−0.08	0.03	0.01
Extraversion	−0.34	−0.11	−0.34	0.03	0.14	−0.08	−0.06	−0.44	−0.22	−0.33	−0.04	0.17	−0.13	−0.09
Honesty-humility	−0.18	0.06	−0.01	−0.32	−0.27	−0.03	−0.27	−0.06	0.07	0.02	−0.20	−0.31	0.11	−0.19
Openness	−0.09	0.05	−0.32	−0.12	−0.05	−0.17	−0.21	−0.11	−0.08	−0.33	−0.13	−0.06	−0.05	−0.20
Psychopathy	0.38	−0.05	0.08	0.22	0.22	0.06	0.22	0.25	−0.05	0.11	0.01	0.19	0.03	0.24
Machiavellianism	0.16	−0.04	−0.03	0.24	0.15	0.01	0.21	0.13	−0.03	−0.04	0.05	0.12	−0.01	0.16
Narcissism	0.00	0.00	−0.06	0.14	0.14	0.03	0.17	0.00	−0.03	−0.08	0.00	0.10	0.00	0.16
Core self-evaluations	−0.08	−0.07	−0.08	−0.07	0.01	0.01	−0.06	−0.18	−0.07	−0.14	0.01	0.10	0.00	−0.01
Competitive worldviews	0.04	−0.16	−0.01	0.25	0.27	−0.19	0.23	−0.04	−0.17	−0.04	0.04	0.20	−0.25	0.14
Self-monitoring	−0.16	0.12	0.02	0.23	0.10	−0.06	−0.03	−0.21	0.12	−0.01	0.22	0.12	−0.13	−0.09

*N = 71. HON SP, Honest self-promotion; HON ING, Honest ingratiation; HON DEF, Honest defensive IM; SIC, Slight image creation; EIC, Extensive image creation; DEC ING, Deceptive ingratiation; IP, Image protection. Values ≥ |0.30| are significant at p < 0.01 (two-tailed), values ≥ |0.28| are significant at p < 0.01 (one-tailed), values ≥ |0.23| are significant at p < 0.05 (two-tailed), and values ≥ |0.20| are significant at p < 0.05 (one-tailed)*.

### Individual differences associated with IM use

#### Honest IM use

When examining the correlations between individual differences and the average IM use across all interviews, we found some significant correlations for honest IM tactics (see Table 2). We note that we used one-tailed significance levels to test all our formal hypotheses (given that they are directional hypotheses), and two-tailed significance levels to explore non-hypothesized relationships. More specifically, we found a positive relationship between Agreeableness and honest defensive IM (*r* = 0.19, *p* < 0.05), providing some support for H1b. In support for H1c, Extraversion was significantly associated with honest self-promotion (*r* = 0.34, *p* < 0.01), as well as honest ingratiation (*r* = 0.20, *p* < 0.05). Consistent with H1d, we found a positive relationship between core self-evaluations and honest self-promotion (*r* = 0.20, *p* < 0.05). However, contrary to H1a, we did not find the expected positive relationships between honest IM and Conscientiousness.

#### Deceptive IM use

We found a number of significant relationships in the expected direction for deceptive IM tactics. This was particularly true for slight and extensive image creation, which were negatively associated with Conscientiousness (*r*s = −0.31 and −0.34, *p* < 0.01 respectively; supporting H2a) and Honesty-Humility (*r* = −0.39, *p* < 0.01 and *r* = −0.25, *p* < 0.05; supporting H2c), but positively associated with Machiavellianism (*r*s = 0.43 and 0.31, *p* < 0.01; supporting H2e), Narcissism (*r*s = 0.26, *p* < 0.01 and 0.23, *p* < 0.05; supporting H2f), Psychopathy (*r*s = 0.37 and 0.30, *p* < 0.01; supporting H2g), and Competitive Worldviews (*r*s = 0.45 and 0.39, *p* < 0.01; supporting H2i). We also found significant relationships for deceptive ingratiation, more specifically with Honesty-Humility (*r* = −0.36, *p* < 0.01; supporting H2c), Extraversion (*r* = 0.21, *p* < 0.05, supporting H2d), and Competitive Worldviews (*r* = 0.22, *p* < 0.05; supporting H2i). We also found significant relationships for image protection with Conscientiousness (*r* = −0.20, *p* < 0.05; supporting H2a), Honesty-Humility (*r* = −0.30, *p* < 0.01; supporting H2c), Extraversion (*r* = 0.20, *p* < 0.05, supporting H2d), Machiavellianism (*r* = 0.26, *p* < 0.05; supporting H2e), Narcissism (*r* = 0.23, *p* < 0.05; supporting H2f), and Competitive Worldviews (*r* = 0.30, *p* < 0.01; supporting H2i). Interestingly, years into the degree program were negatively correlated with three forms of deceptive IM (*r*s = −0.21 to−0.26), such that students at earlier stages of their education reported using more deceptive IM. Finally, we did not find any relationship for Agreeableness or self-monitoring with any of the forms of deceptive IM, and thus H2b and H2h were not supported.

### Individual differences associated with variability in IM use

The correlations between individual differences and the two indicators of variability of IM use demonstrate a similar pattern of results, although we found more significant relationships when variability was measured with the standard deviation (see Table 3).

#### Variability in honest IM use

With the standard deviation indicator, Psychopathy was positively associated with variability in honest self-promotion (*r* = 0.38, *p* < 0.01), providing some support for H3f. Although these variables were not associated with any hypotheses, we found Extraversion to be negatively associated with variability in honest self-promotion (*r* = −0.34, *p* < 0.01) and honest defensive IM (*r* = −0.34, *p* < 0.01), and Openness to be negatively associated with variability in honest defensive IM (*r* = − 0.32, *p* < 0.01).

When variability was measured with the coefficient of variation, Psychopathy was also positively related to variability in honest self-promotion (*r* = 0.25, *p* < 0.05), further supporting H3f. However, and contrary to H3a, self-monitoring was negatively associated with variability in honest self-promotion (*r* = −0.21, *p* < 0.05). Moreover, we found similar relationships for Extraversion and Openness as we did with the standard deviation indicator. More precisely, we found Extraversion to be negatively associated with variability in honest self-promotion (*r* = −0.44, *p* < 0.01) and honest defensive IM (*r* = −0.33, *p* < 0.01), and Openness to be negatively associated with variability in honest defensive IM (*r* = −0.33, *p* < 0.01).

#### Variability in deceptive IM use

With the standard deviation indicator, self-monitoring was positively associated with variability in slight image creation (*r* = 0.23, *p* < 0.05), providing some support for H3a. Competitive Worldviews was positively associated with variability in slight image creation (*r* = 0.25, *p* < 0.05), extensive image creation (*r* = 0.27, *p* < 0.05), and image protection (*r* = 0.23, *p* < 0.05), thus providing support for H3b. Machiavellianism was positively associated with variability in slight image creation (*r* = 0.24, *p* < 0.05) and image protection (*r* = 0.21, *p* < 0.05), providing some support for H3d. In line with H3f, Psychopathy was positively associated with variability in slight image creation (*r* = 0.22, *p* < 0.05), extensive image creation (*r* = 0.22, *p* < 0.05), and image protection (*r* = 0.22, *p* < 0.05). Honesty-Humility was negatively associated with variability in slight image creation (*r* = −0.32, *p* < 0.01), extensive image creation (*r* = −0.27, *p* < 0.05), and image protection (*r* = −0.27, *p* < 0.05), providing support for H3g. Finally, although it was not associated with any hypotheses, we found Conscientiousness to be negatively associated with variability in extensive image creation (*r* = −0.27, *p* < 0.01). However, we found no significant relationship for core-self-evaluations and Narcissism, thus failing to provide any support for H3c and e.

When variability was measured with the coefficient of variation, several of these relationships continued to be in line with our hypotheses. For instance, Psychopathy was positively related to variability in image protection (*r* = 0.25, *p* < 0.05), providing some additional support for H3f. Moreover, Honesty-Humility was negatively related to variability in slight (*r* = −0.20, *p* < 0.05) and extensive image creation (*r* = −0.31, *p* < 0.05), thus providing some additional support for H3g. In line with H3a, self-monitoring was positively related to variability in slight image creation (*r* = 0.22, *p* < 0.05). Results for Competitive worldviews provided more inconsistent evidence. In line with H3b, Competitive worldviews were positively related to variability in extensive image creation (*r* = 0.20, *p* < 0.05), but also negatively with variability in deceptive ingratiation (*r* = −0.25, *p* < 0.05). Moreover, we found no significant relationship for core-self-evaluations, Narcissism, or Machiavellianism. We provide a summary of all supported vs. unsupported hypotheses in Table 4.

**Table 4:** **Summary of supported vs. unsupported hypotheses**.

	**Hypothesis**	**Supported**	
H1a	Conscientiousness is positively associated with honest IM use	No	
H1b	Agreeableness is positively associated with honest IM use	Yes	
H1c	Extraversion is positively associated with honest IM use	Yes	
H1d	Core self-evaluations are positively associated with honest IM use	Yes	
H2a	Conscientiousness is negatively associated with deceptive IM use	Yes	
H2b	Agreeableness is negatively associated with deceptive IM use	No	
H2c	Honesty-Humility is negatively associated with deceptive IM use	Yes	
H1d	Extraversion is positively associated with deceptive IM use	Yes	
H2e	Machiavellianism is positively associated with deceptive IM use	Yes	
H2f	Narcissism is positively associated with deceptive IM use	Yes	
H2g	Psychopathy is positively associated with deceptive IM use	Yes	
H2h	Self-monitoring is positively associated with deceptive IM use	No	
H2i	Competitive Worldviews are positively associated with deceptive IM use	Yes	
		*SD*	*CV*
H3a	Self-monitoring is positively associated with variability in IM use	Yes	Inc.
H3b	Competitive Worldviews are positively associated with variability in IM use	Yes	Inc.
H3c	Core self-evaluations are positively associated with variability in IM use	No	No
H3d	Machiavellianism is positively associated with variability in IM use	Yes	No
H3e	Narcissism is positively associated with variability in IM use	No	No
H3f	Psychopathy is positively associated with variability in IM use	Yes	Yes
H3g	Honesty-Humility is negatively associated with variability in IM use	Yes	Yes

*SD, Results with the Standard Deviation indicator; CV, Results with the Coefficient of Variation indicator; Inc., Inconsistent results for honest vs. deceptive IM*.

## Discussion

### Contribution to IM research

The present research examined the antecedents of IM use and variability in IM use, and contributes to the IM and personnel selection literature in several ways. First, this study explores a large number of individual differences and their relationship with both honest and deceptive IM use. Our results were largely aligned with theoretical propositions from IM models (Levashina and Campion, 2006; Roulin et al., 2016). Our study thus helps clarify findings from earlier research, which have examined IM without distinguishing honest from deceptive tactics (Kristof-Brown et al., 2002; Higgins and Judge, 2004) or focused on deceptive IM only (Levashina and Campion, 2007; Roulin and Krings, 2016). It also replicates recent findings with both honest and deceptive IM (Bourdage et al., 2015) and complements them by including additional applicant characteristics (e.g., the dark triad of personality and core self-evaluations). Importantly, it is the first study that relies on a more robust and reliable approach to measure IM, by using the average IM use across multiple interviews (with M_number interviews_ = 5.80) instead of just one interview.

#### Antecedents of honest vs. deceptive IM use

Overall, our results confirm that only very few individual characteristics are associated with honest IM use. Honest IM users tend to me more extraverted and have stronger core self-evaluations. However, our findings highlight a very different profile for applicants who engage in deceptive IM. Those who use more deceptive IM tactics are less experienced, lower on Conscientiousness and Honesty-Humility, but higher on Extraversion, the dark triad of personality (i.e., Machiavellianism, Narcissism, and Psychopathy) and Competitive Worldviews. Overall, such findings are in line with previous studies (Levashina and Campion, 2007; Hogue et al., 2013; Roulin and Krings, 2016) and provide additional evidence supporting the practical importance of distinguishing between honest and deceptive IM (Levashina et al., 2014), and the claims that deceptive IM could be detrimental for organizations (Levashina and Campion, 2006).

Indeed, many of the antecedents of deceptive IM, such as low Conscientiousness (Barrick and Mount, 1991), low Honesty-Humility (Lee et al., 2005), or high scores on the dark triad (O'Boyle et al., 2012) are also associated with lower work performance or increased likelihood of engaging in counterproductive work behaviors. In other words, applicants who fake in interviews likely possess a personality profile that is undesirable for employers. From a practical perspective, the smaller dispositional basis of honest IM indicates that the information contained in honest IM, such as job-relevant qualifications and fit information, will not just be exhibited by Conscientious, qualified applicants, but that rather interviewers may need to draw this information out from candidates. Future research should therefore focus on how situational factors (e.g., the use of various question types) or target factors (e.g., characteristics or behavior of the interviewer) can impact the use of honest and deceptive IM.

#### Antecedents of variability in IM use

This study is the first to examine the variability of IM use across multiple interviews. It thus represents an initial attempt to explore the dynamic and adaptive nature of applicant IM (Roulin et al., 2016). Our findings suggest that some IM tactics are more stable, whereas others are more variable (or prone to adaptations). For instance, other-focused tactics like ingratiation and defensive tactics appear to be more variable than self-focused tactics like self-promotion or extensive image creation. This is in line with the research suggesting that some interview formats or types of questions can facilitate or impede IM use (Van Iddekinge et al., 2007; Levashina et al., 2014). Indeed, ingratiation tactics are oriented toward the organization (i.e., praising the company's accomplishments, highlighting the fit between ones' values and those of the company) or the interviewer (e.g., flattery, highlighting similarity), which may be more or less difficult to use depending on the individual in charge of conducting the interview and the organization one is applying for (Chen et al., 2008). Similarly, defensive IM are reactive tactics used to protect or repair one's image (Tsai et al., 2010). The necessity to use defensive IM tactics thus likely depends on the questions asked by the interviewer, with some interviewers asking more difficult (or failure-oriented) questions than others.

In line with the notion that applicants' use of ingratiation or defensive IM tactics is largely contingent on the target and situation, significant relationships between the variability indicators for those particular IM tactics and individual differences were rare in this study. In contrast, variability in self-promotion and slight or extensive image creation was more strongly associated with individual differences. Roulin et al. (2016) suggested that high self-monitors are more able to learn from past interviewing successes and failures, thus increasing their capacity to use deceptive IM from one interview to the next. They also suggested that individuals with an internal locus of control and more competitive views of the world tend to attribute failures to the competition for the job, and are thus more likely to adapt their motivation to use IM in subsequent interviews. In line with these predictions, we found that self-monitoring and Competitive Worldviews were positively associated with variability in the use of image creation tactics, although the effects were clearer when IM variability was captured with the standard deviation indicator. This confirms that applicants who describe themselves as able to effectively manage their behaviors and evaluate others' reactions to such behaviors, and those who perceive the world to be a competitive jungle, are more likely to adapt their faking strategy from one interview to the next. However, we did not find any associations between core self-evaluations (which incorporate internal locus of control) and variability in IM use.

One explanation for these results could be that, in the present study, applicants were mostly involved in multiple interviews over a short period of time (i.e., about 2 weeks). They were thus forced to adapt their IM strategy based on their perceptions of success, and before being informed about the actual interview outcomes. Locus of control is arguably more relevant when applicants are informed about the interview outcome and have to interpret it (e.g., attribute their failure to internal or external causes) before deciding how to adapt their behaviors. Similarly, we would argue that the effects for Competitive Worldview and self-monitoring may also have been stronger if applicants had been informed of their performance before their next interview or had had more time to reflect on their behaviors and performance. Reflection and feedback would have offered applicants more opportunities to interpret their failure as caused by competition or to learn from their interviewing experience, which is necessary to be able to optimize one's IM strategy.

We also found some relationships in the expected direction for the dark triad of personality and Honesty-Humility, as well as negative relationships between variability in using image creation tactics and Conscientiousness. In other words, applicants who are less conscientious, less honest, and who possess higher levels of “dark” traits (especially for Psychopathy) also tend to adapt their use of deceptive IM across interviews. This is consistent with past research suggesting that such individuals are naturally more willing to take risks and adapt their behavior to better influence others (Jonason and Schmitt, 2012; Jonason et al., 2012). In the interview context, this implies adapting their IM tactics, such as exaggerations or inventions, to increase their chances of getting the job. Moreover, it may imply that individuals with these characteristics will be more or less likely to fake in certain interview situations or with certain types of interviews, an interesting direction for future research. Additionally, we observed negative relationships between Extraversion and variability in honest IM use (mostly for self-promotion and honest defensive IM). Because IM requires social or interpersonal skills (Levashina and Campion, 2006; Roulin et al., 2016), it may be that introverted applicants simply do not have the capacity to adapt and engage in more IM.

#### A clearer profile of IM users

Our results about IM variability become even more pertinent when coupled with the findings for IM use described above. For instance, they suggest that honest and humble applicants initially refrain from using IM tactics and tend to adhere to this modest approach throughout all their interviews. In contrast, those who possess a “darker” personality profile tend to use more deceptive IM and are more likely to adapt their IM strategy across multiple interviews in order to increase their chances of success. An applicant with a “darker” personality may thus be more willing to try different tactics in different interviews. For instance, an applicant with such a profile may have used honest IM in an initial interview, perceived this strategy to be ineffective, and may be willing to try using more deceptive IM in subsequent interviews (whereas a more a honest and humble applicant may not).

If individuals with a “darker” personality are able to adapt their behaviors and effectively use deceptive tactics adjusted to each situation, this may explain why fakers can sometimes obtain higher evaluations from interviewers (Levashina and Campion, 2007). On the one hand, this capacity to adapt one's IM behavior to the situation could be seen as a valuable skill, which is consistent with claims that IM can have some construct-related validity (Kleinmann and Klehe, 2010). On the other hand, because applicants who are better at adapting their IM use are also those who possess undesirable personality profiles (Lee et al., 2005; O'Boyle et al., 2012), valuing such skills could be risky for organizations. Moreover, individuals high on Psychopathy in particular were also more likely to vary their use of honest self-promotion, indicating these very undesirable individuals may engage in an honest IM behavior if the situation calls for it, or perhaps if they believe it will be successful.

### Limitations and future research directions

This research has some limitations and associated avenues for additional research. First, although our study is based on IM data from a total of 448 real interviews with professional interviewers, at the applicant level our sample size is somewhat small (i.e., *N* = 80 for IM use, and *N* = 71 for IM variability). We observed a number of additional non-significant correlations that were in the expected direction and with effect sizes in the range of previous studies (i.e., 0.15–0.25), which would possibly have emerged as statistically significant with a larger sample size. Future research could thus try to replicate our findings with a larger number of applicants. Studies with larger samples would also open the door to additional analyses, such as exploring more complex patterns of behaviors across interviews. As an example, future research could explore if applicants with darker personality traits engage in increasingly more deceptive tactics.

Second, the variability in IM use (and some relationships between individual differences and IM variability) may have been reduced by the unique characteristics of the interview process used in this study. As described above, applicants were involved in multiple interviews over a 2-week period, and they likely did not receive any formal feedback about their performance until they completed all their interviews. Applicants were thus forced to adapt their IM behaviors from one interview to the next based only on their perceived performance, which is likely a weaker feedback mechanism than being informed of the actual interview outcome by the organization. For instance, some applicants may have inaccurately perceived their performance in the initial interviews to be satisfactory, and thus refrain from subsequently adapting their use of IM. The same applicants may have engaged in more adaptations after having received actual feedback. Future field studies could follow applicants on the job market over a longer period of time in order to insure that they receive feedback about their performance between interviews. These studies should incorporate direct measures of the actual success of applicants in the interview (or, at least, applicants' perceptions of success) to further understand what drives adaptations in IM. Alternatively, experimental designs could be used to manipulate the feedback provided to applicants and observing changes in IM use in a subsequent interview.

Third, our study is only an initial (and thus imperfect) attempt to test the adaptations in IM use described in Roulin et al.'s (2016) model. More precisely, we measured the variability in IM use as a proxy for applicants' adaptations, but we did not examine the mechanisms driving those adaptations. For instance, we did not examine if changes in behaviors were caused by changes in applicants' capacity and/or motivation to use IM. Future research could attempt to use a more complex longitudinal approach and measure the motivation and capacity to use IM prior to interviews, use of IM during interviews, and interview outcomes for a number of consecutive interviews.

Finally, our study focused on individual differences as antecedents of IM use and IM variability. Yet, both our results for other-focused and defensive IM tactics and previous research (e.g., Van Iddekinge et al., 2007) suggest that situational factors may also play an important role. Future studies could examine the combined impact of individual differences and situational factors on IM use and adaptations. We would especially urge researchers to investigate the role of interviewers' (i.e., target) characteristics. The target of influence tactics is an important factor in workplace IM research (Ferris and Judge, 1991), but has largely been overlooked in interview IM research, which has mostly focused on the content of the interview (e.g., level of structure or question type).

## Conclusion

Earlier IM research has highlighted that applicants' behavior in an employment interview is a complex phenomenon, driven by an interaction of individual difference and contextual factors (e.g., Levashina and Campion, 2006). Moreover, recent theoretical advances in personnel selection have suggested that applicant IM should be considered as the outcome of a dynamic and adaptive process (Bangerter et al., 2012; Roulin et al., 2016). The present study was built on those foundations and examined the role of individual differences in applicants' use of IM tactics and adaptations in IM behaviors across multiple interviews. Our findings particularly emphasized the important role of stable traits like low Honesty-Humility, the dark triad of personality, self-monitoring, or Competitive Worldviews in using and adapting deceptive IM strategies. Individuals possessing such traits are thus not only impression managers (or fakers) on one occasion, but likely to be flexible impression managers (or fakers) who adapt their behavior across interviews. Moreover, because these traits are associated with undesirable workplace outcomes, individuals possessing them may represent a threat for organizations.

## Author contributions

Both co-authors contributed to the study preparation, the study design, the development of the theoretical foundations for the manuscript, and the manuscript preparation and writing. NR was also in charge of the data collection, the data analyses, preparation of tables, and references.

## Funding

This project was supported by an Insight Grant from the Social Science and Humanities Research Council of Canada (Grant # 435-2015-0566) awarded to NR (Principal Investigator) and JB (Co-Investigator).

### Conflict of interest statement

The authors declare that the research was conducted in the absence of any commercial or financial relationships that could be construed as a potential conflict of interest. The reviewer MB and handling Editor declared their shared affiliation, and the handling Editor states that the process nevertheless met the standards of a fair and objective review.
